# Drug shortages in Israel: regulatory perspectives, challenges and solutions

**DOI:** 10.1186/s13584-017-0140-9

**Published:** 2017-04-03

**Authors:** Eyal Schwartzberg, Denize Ainbinder, Alla Vishkauzan, Ronni Gamzu

**Affiliations:** 1 Pharmaceutical Division, Ministry of Health, 39 Yirmiyahu St, Jerusalem, Israel; 2 Ben-Gurion University of the Negev, Beersheba, Israel; 3 Tel Aviv Sourasky Medical Center, Tel Aviv, Israel

**Keywords:** Drug shortage, Supply, Quality, Deficiency, Medication, Well-established, Marketing withdrawal, Temporary, Permanent, Notification, Pharmaceutical, Prescription, Pricing

## Abstract

**Background:**

Pharmaceutical drug shortages (DSs) are a global problem which presents challenges to countries around the world. Shortages of pharmaceutical products may have a direct detrimental impact on public health and patients’ wellbeing by causing delayed, or even lack of, treatment. Moreover, DSs may force both patients and caregivers to use unfamiliar drugs, which could lead to medication errors. The objective of our study was to analyze DSs in Israel during the years 2013–2015, assessing their etiology and exploring the steps taken for their mitigation and prevention.

**Methods:**

The database of the Israeli Ministry of Health (MoH) on drug shortages contains all the DSs recorded in Israel since 2013, detailing the cause of the DS, its duration, steps taken in its’ management and the availability of generic or therapeutic alternatives. Selected examples of DSs from the database are described in this paper in order to identify the causes of DSs, the scope of the problem and possible solutions. Additionally, we have reviewed the recent activities performed by European Medicine Agency (EMA) and the American Food and Drug Administration (FDA) in their efforts to minimize this problem.

**Results:**

Several factors contributing towards DSs in Israel were identified, including quality problems in both the final drug product and in the raw materials, upgrades and improvements of the manufacturing process required by the MoH, manufacturing by a sole supplier, dramatic price decrease in off-patent medications causing the manufacturer to discontinue the distribution of the product in Israel, just-in-time inventory control, and others.

One of the most important steps in managing drug shortages was identified to be early notification of the shortage by the Marketing Authorization Holder (MAH) to the MoH. In 2013, the Israeli MoH updated the regulation on drug shortages instructing MAHs on their obligation of early notification to the MoH.

Furthermore, various steps dealing with marketing withdrawal of drugs and temporary drug shortages are being implemented in Israel, such as suspending any further reductions in drug prices below 17 new Israeli shekels, instructing all MAHs to maintain no less than 1 month supply of all registered and non-registered drugs in Israel and allowing an expedited registration pathway for well-established use/grandfather drugs.

**Conclusions:**

Drug shortages pose significant public health hazards worldwide. Early notification to the MoH and open dialog with MAHs are essential for managing DSs and mitigating their impact. Despite the efforts carried out by health regulatory authorities worldwide, DSs still pose a significant threat to public health.

## Background

A drug shortage (DS) is defined by the Food and Drug Administration (FDA) as a situation when: “the total supply of all clinically interchangeable versions of a FDA-regulated drug product is inadequate to meet the current or projected demand at the patient level” [1].

DSs are a global problem relevant to countries regardless of size. In the United States (US), the number of prescription DSs more than tripled between 2005 and 2010, increasing from 61 in 2005 to 178 in 2010 [2]. This significant increase in the number of drugs in shortage resulted in several actions taken by the US government in order to address the problem. An executive order signed by the US President in 2011 and the FDA Safety and Innovation Act (FDASIA) passed in 2012, both aimed to expand the authority of FDA in managing DSs and to ensure advanced notification of anticipated shortages by Marketing Authorization Holders (MAH) to the FDA. Nevertheless, in spite of these efforts and their relative success in preventing DSs, announcements on new shortages appear on the FDA website almost every week.

As an additional step in coping with the DS challenge in the US, the FDA recently established the Drug Shortage Assistance Award program aiming to provide public recognition to drug companies and manufacturers who have worked in cooperation with the FDA and have implemented strategies to help ensure a steady supply of necessary drugs. Award letters posted on the FDA website recognize these companies for making a substantial contribution to preventing or alleviating a DS. Advocates of this initiative hope that these public letters will serve as an incentive for other companies to maintain a stable drug supply and avoid DSs by working together with the regulator.

Furthermore, a publicly available database of DSs was established on an FDA website, keeping all the stakeholders updated on new entries and resolved shortages [3].

Data from the European Union (EU) is relatively lacking compared to the US and most DSs are dealt with at a national level. Nevertheless, the European Medicine Agency (EMA) is concerned with DSs as well, focusing mainly on shortages associated with quality defects affecting many EU countries. In November 2012, the EMA published a position paper on medicinal product supply shortages caused by manufacturing problems [4]. Subsequently, an implementation plan was issued in order to alleviate the increasing number of unforeseen DSs at a European Community level [5].

In 2014 the Organization of Economic Co-operation and Development (OECD) released a report from the Global Forum on Competition that focused on the distribution of pharmaceuticals [6]. Shortages in some OECD countries were described. The shortage problem was not confined to countries with severe financial crises such as Greece and Spain. The UK, for example, has suffered from systemic shortages and supply disruptions, mainly due to EU regulations allowing free movement of medicines within the EU (parallel trade) thereby impacting the balance of individual country supply and demand. UK authorities have responded by introducing legislation requiring pharmacists to acquire a wholesale license before they can engage in any parallel trade. They also established a requirement for the wholesalers, as their first responsibility, to maintain an appropriate and continued supply of medicinal products to pharmacies within UK so that first the needs of local patients are covered. Breach of this regulation, set by the MHRA, may lead to regulatory action against the involved wholesaler, resulting in the revoking of the pharmaceutical license and even criminal prosecution.

The Business and Industry Advisory Committee to the OECD (BIAC) has suggested that the economic crisis has forced deep price cuts to pharmaceutical products, as governments have attempted to reduce healthcare spending [7]. The causes of DSs, in their view, included globalization of manufacturing and distribution activities, leading to fewer supply sites and thus less flexibility; scale of demand, as well as pricing and reimbursement policies.

At the beginning of 2016, the World Health Organization (WHO) released a new report on global drug shortages [8]. The WHO commented that while DSs are not a new phenomenon, they have been increasing in recent years, resulting in increased worldwide concern regarding the supply of essential medicines.

Shortages of pharmaceuticals can result in delayed treatment for patients and sometimes even deprivation of treatment due to lack of availability of the needed drug. When no appropriate alternative therapy is available, DSs could lead to a serious threat to public health.

Moreover, DSs are often the cause of medication errors. Alternative drugs, unfamiliar to clinicians or patients, different brands or concentrations, altered presentations, all may cause medication errors, adverse events and decreased patient adherence.

A recent paper by CL Ventola, emphasized the consequent mortality associated with DSs in the US [9]. For example, a shortage of a life-supporting oncological drug could mean a death sentence to patients in need. In a survey carried out in 2012 in the US among pharmacy directors from various sectors, including acute care, non-acute care, management, and industry, 40% of the participating hospital pharmacies (with more than 85% of the responders from acute care hospitals) reported adverse events associated with DSs. DSs were also found to be associated with numerous medication errors, including drug omission, wrong dose and drug administrations. Many responders to the survey reported that DSs lead to delayed or even cancelled care. These cancellations included procedures, surgeries, chemotherapy and other treatments [10].

Numerous studies and reviews have referred to the possible impact of DSs on public health. Exploring the problems and the negative outcomes of DSs, Clauder et al. have conducted a survey among pharmacy directors in North Carolina, South Carolina, Georgia, and Florida [11]. Responders reported that DSs cause 1 to 5% error rates in hospitals, and 60% of the time DSs create unsafe conditions for patients and staff. In a survey carried out in a pediatric Intensive Care Unit (ICU), surprisingly, no increase in medication error rate was found due to shortage of sedatives. The authors hypothesize that the reason for this lack of difference is ongoing communication and information provided by the clinical pharmacists and routine face-to-face education with prescribers during the shortage period [12].

Besides affecting the quality and the safety of public health, DSs have a significant financial effect on health systems. For example, the financial burden of drug supply disruptions to hospitals in the US was estimated in 2011 to be $200 million annually, because of the need to purchase more expensive therapeutic substitutes. Another $216 million of indirect costs were estimated as a result of labor costs due to time spent by healthcare personnel in the management of DSs in hospitals [9, 13]. These estimations take into account only part of the financial burden of DSs, as they neglect the costs of incorrect or non-optimal treatment, leading to an increased number of hospitalization days, additional drugs required, etc. Nearly all responders to a survey carried out in hospitals and other healthcare institutions in 2010 in the US experienced an increase in drug costs as a result of a shortage, due to purchasing drugs off-contract, buying more costly brand names and alternative medications at increased price due to limited supplies [14].

In this article, we will describe the current situation in Israel with reference to other countries emphasizing special circumstances relevant to the Israeli health system. Due to the lack of data on outpatient DSs worldwide, the analysis of the situation in Israel, and references to EMA and FDA, focuses mainly on inpatient DSs. Furthermore we will discuss approaches and solutions to overcome this phenomenon.

## Methods

Since 2013, The Israeli Ministry of Health (MoH) receives and documents notifications regarding supply shortages from MAH. We have searched this database to identify the scope of the problem and steps taken by the Pharmaceutical Division in the prevention and management of DSs. Furthermore, recent policies by EMA and FDA in the management of DSs are described.

## Results and discussion

### Drug shortages in Israel

Based on the data collected by MoH, between 2013 and 2015, 677 DS notifications were received: 191 in 2013, 240 in 2014 and 246 in 2015. In the US, 117, 44 and 44 DSs were tracked in the years 2012, 2013 and 2014, respectively. The FDA helped to prevent 282 DSs in 2012, 170 shortages in 2013 and 101 shortages in 2014 [3]. Of these DSs, 31% had no available therapeutic alternatives. Sixty to seventy percent of the DSs were communicated by MAH to MoH at the time of DS coming into effect or up to 1 month before, lacking advanced notification essential for management and prevention of DSs. These shortages included sole supplier drugs for various medical conditions, including tranexamic acid tablets and injection, cefotaxim sodium injection, fluphenazine decanoate injection, metolazone tablets, ipatropium bromide respiratory solution, nitroprusside solution, clomifene citrate tablets, oral midazolam, acyclovir ophthalmic ointment, alfacalcidol drops, barium sulfate suspension for radiography, morphine injections, lidocaine injections, midazolam injections, melphalan injections, adrenalin injections, etc.

**Fig. 1:**
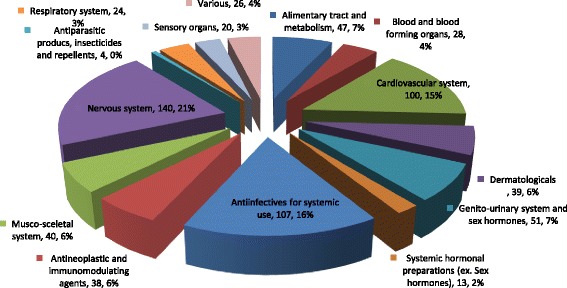
Distribution of drug shortage notices by ATC categories in Israel during 2013–2015

Figure 1 demonstrates the distribution by ATC (Anatomical therapeutic chemical) categories of DSs in Israel during the years 2013–2015. DSs were disseminated throughout a wide array of the therapeutic groups and affected various aspect of medical care. The most prevalent ones included cardiovascular drugs (15%), anti-infectives for systemic use (16%) and drugs for the treatment of nervous system disorders (21%).

The following case studies provide examples of the complexity of DSs as seen in Israel, some of which may be relevant to other countries as well.

At the beginning of May 2013, the Israeli MoH received a notice of a possible shortage of phenytoin effective almost immediately and anticipated to last for a period of up to 6 months. Phenytoin is an anti-epileptic drug with narrow therapeutic range and non-linear metabolic elimination. Its therapeutic range is so limited, that cases of patients developing breakthrough seizures due to batch-to-batch variations are described in the scientific literature [15]. In dealing with this potential shortage, a careful assessment and risk minimization measures are paramount. In a small country like Israel, where only one drug supplier of phenytoin exists, no local alternatives are available. Attempts by the MAH to find another source of drug supply were futile since the shortage was global. A special task force, including the four Health Management Organizations (HMOs) and leading expert neurologists was established by the Israeli MoH, in order to address this shortage. Due to the narrow therapeutic index of phenytoin, it was important to ensure that the alternative will resemble as much as possible the currently supplied drug to minimize the risk of patients developing breakthrough seizures. In collaboration with the Israeli Neurology Association, a decision was made to supply the remainder of the drug to patients already receiving phenytoin; directing new patients towards alternative therapies. Several potential candidates for phenytoin generics were located in Europe. Since no studies comparing the various phenytoin products were found, it was decided by the task force that comparative bioavailability tests should be carried out to determine which of the available alternatives most resembles the product registered and marketed in Israel. Fortunately, at the last minute, the MAH was able to find an alternative supply site for the original drug and the threat of DS was addressed.

Another example of a DS is Clomiphene citrate, a sole supplier drug used for induction of ovulation. Following intervention by the MoH, the MAH was able to allocate to the Israeli market a supply of the same drug manufactured in Israel for export, thus temporarily resolving the shortage.

Additional imminent shortage of a topical preparation for treatment of burn wounds arose following rapid alert report received by the MoH in 2014 regarding possible cross-contamination with penicillin. Following risk assessment of drug shortage vs. possible side-effect as a result of cross-contamination, it was decided to add a special labeling to the drug’s package notifying the customers that this product should not be used in patients sensitive to beta-lactams. The distribution of the product was accompanied by a Dear Health Care Provider (DHCP) letter and a notice to the public made by MoH.

In March 2014, a routine inspection of the manufacturing facility of oromucosal solution of midazolam, a drug indicated to stop sudden and prolonged convulsive seizures in children, was carried out. The inspection revealed failures in the quality management of the manufacturing process of the drug, raising concerns regarding a potential risk of cross-contamination with amsacrine, another medicine produced at the same site used for the induction and maintenance of remission in acute leukemia in adults. Tests did not find any residuals of amsacrine. As a precautionary step, the drug was recalled in Member States where suitable alternative treatments were available. Due to the lack of available alternatives, the product was classified as critical and it was decided to maintain its supply to the Israeli market.

One of the major concerns in DSs worldwide is disruption in the supply of sterile injectables, resulting in severe impact on public health. During the years 2013–2015 in Israel, 27% of DSs were supply interruptions of injectables and approximately 20% of them were IV injections. Surprisingly, this was in contrast to the situation in the US, where a significantly higher incidence of shortages related to sterile injectable products was noted. For example, according to FDA data in years 2011–2012, sterile injectables accounted for about 70% of all DSs [16].

Throughout 2014–2015, Israel experienced three major DS crises leading to disruption of drug supply; two of them involved local manufacturing sites. In one case, all the production of a MAH was moved to a new manufacturing plant. During the preparation for this major variation, following discussions between the MoH and the MAH, it was decided to increase the inventory of critical drugs to 6 months of use, taking into account the MAH being the sole supplier for these products. Unfortunately, due to unexpected production difficulties at the new facility, these efforts were insufficient, resulting in an immediate shortage of many critically important injectables such as midazolam, lidocaine and morphine. In another incident, one of the major pharmaceutical companies in Israel relocated the secondary packaging site of solid oral dosage form products, creating an ongoing temporary shortage in 90 of the company’s drugs (some of them with a single supplier). Another crisis, related to the transfer of the production of injectables to a new manufacturing facility abroad, is still having an impact on their supply chain, resulting in the need to import non registered alternatives. The MoH is working closely with the MAH, other manufacturers, importers and healthcare institutions to find a solution for this problem.

### Etiology of drug shortages in Israel

The etiology of DSs in Israel is similar to FDA and EU, being multifactorial and related to one or more of the following factors:

- Quality problems that are not limited to the final drug product, but often found in the raw materials, affect drug production and supply around the globe. Major challenges are quality problems with Active Pharmaceutical Ingredient (API) from emerging countries like India and China. Just recently, Good Manufacture Practice (GMP) approvals of several large global manufacturers of APIs have been revoked following inspections carried out by mature regulatory authorities. Failure of quality management in facilities that produce the finished dosage form of the drug was directly responsible for 56% of sterile injectable DSs in 2011 in the US [17].
- Disruption or delay in the supply of the raw or bulk material can affect multiple manufacturers and result in a country/regional/worldwide DS. For example, a shortage of the injectable antibiotic streptomycin was reported in 15 countries in 2010, with 11 more countries predicting their stock would run out before they could be replenished [18].
- Regulatory authorities’ requirements for improvement of the manufacturing process have become a complex and expensive effort for pharmaceutical companies. Many companies, especially, but not limited to small ones, are unable or unwilling to make capital investments to sufficiently upgrade the infrastructure required according to the updated GMP standards. Hence, manufacturers often choose to discontinue production of low priced off-patent drugs. The phenomena is further accentuated in a small country with strict regulations like Israel, where local manufacturers are sometimes required to implement changes in the manufacturing/validation process of drugs intended for a relatively small local market.
- The situation in Israel, with limited market size and number of competitors, results in many drugs being manufactured solely by one company. Therefore, in case of manufacturing or supply difficulties, the risk for a nationwide shortage constantly exists. Special emphasis should be drawn to sterile injectable products with revenues barely covering the costs of production. Currently in Israel, numerous old, well established and extensively used drugs in an ICU setting, like lidocaine, midazolam and dipyridamole, are manufactured and registered by a single company.
- Low prices of certain off-patent medicines may contribute to DSs and drive manufacturers of these medicines out of the market. Furthermore, in Israel, reference pricing policy is determined by referencing overseas countries, but not necessarily reflecting global and local market conditions. Fluctuations in currency exchange rates influence prices as well. This, in turn, may lead to situations in which the revenue of drugs will not cover the costs of production, resulting in permanent DSs.
- Just-in-time inventory control by manufacturers, medical product distributors and healthcare institutions reduces the possibility to cope with DSs at times of unexpected demand changes or supply disruption. In cases in which an MAH is the main or sole source for a medication on the market, a manufacturing/supply chain problem can often result in a DS affecting the entire country.
- The drug market in Israel is centralized and dominated by Health Management Organizations (HMOs) with large scale generic medication tenders. Therefore, all approved generics and innovative drugs of the same International Nonproprietary Name (INN) are perceived as switchable by the HMOs (with the exception of biosimilars, where a different policy exists). Thus, the HMOs buy their drugs based almost entirely on pricing. This often leads to a situation, where an MAH that fails to win a tender discontinues manufacturing or local distribution of the drug, resulting in only one alternative with sole supplier on the market.
- The Israeli regulation currently mandates Certificate of Pharmaceutical Product (CPP) from a pre-determined limited set of “authorized countries” as stated in the legislation. This demand is a prerequisite for drug registration. Consequently, this limits the drugs submitted for registration in Israel.

### Steps for managing drug shortages based on the Israeli experience

Unfortunately, without supporting legislation, a regulatory authority cannot instruct a pharmaceutical company/MAH to produce a drug. It also has no capacity to influence a DS that occurs due to commercial agreements between manufacturers and healthcare institutions.

While the root cause of DSs is usually outside the regulator’s control, some of the shortages can be managed and even prevented by activities promoted by the MAH and/or the regulator, especially when both parties collaborate effectively.

One of the key steps in overcoming some of the DSs is ensuring that the regulator receives a notice of the upcoming shortage well in advance. Early disclosure of a shortage by the MAH can allow the regulator to find alternative solutions to overcome the crisis.

In their attempt to prevent and mitigate DSs, the FDA implemented section 506 (c) of the Federal Food Drug and Cosmetic Act requiring MAHs to notify of any manufacturing discontinuance or interruption in manufacturing of the product at least 6 months prior to the date of the discontinuance or as soon as possible and in no case later than 5 business days after DS occurs. EMA requires MAHs to notify the Members State’s competent authority no less than 2 months before the expected interruption in the market.

Until May 2013, the Israeli MoH required MAHs to notify only in cases of permanent marketing termination. The procedure for the notification process in event of drug shortage, also known as Procedure 104, was updated, requiring MAHs to notify the MoH of any permanent or temporary DS anticipated to last more than 2 weeks, at least 6 months prior to the estimated shortage start date. This notification requirement enables the Israeli MoH to monitor DSs in Israel, to receive early notifications of such incidents and to take risk mitigation measures. Additionally the procedure requires the MAH to discuss any future termination with the MoH in order to find acceptable solutions in view of public health considerations. The MoH reserves the right to instruct the MAH to continue distribution of a medicine under special circumstances.

During the years 2013, 2014 and 2015, 58, 71 and 64% (respectively) of the DS notices were received immediately before the shortage (less than 1 month before the shortage came into effect). A decrease in immediate notifications was noted in the months following the update of the procedure. Nevertheless, in spite of the updated requirements, many MAHs still fail to provide the MoH with adequate notice on forthcoming shortages. The lack of compliance with the early notification requirements could be due to the fact that the MAH is not always aware in advance of future shortages and thus is unable to meet the requirement for 6 months notification. This is especially evident when the disruption of the drug supply is caused by global API shortage, quality issues requiring withdrawal from the market of one or more batches of the product, noncompliance with GMP requirements following regulatory inspection and others.

The Israeli MoH developed a protocol for the management of DSs based on country specific experience. According to the protocol, following the MAH notification of the forthcoming shortage of drug with no generic alternatives, the MoH engages all the relevant stakeholders, including Health Care Professionals (HCP), HMOs and/or hospitals and drug importers, to identify a possible solution (e.g. importing similar drug from abroad or issuing instructions to HCPs regarding the shortage and the possible therapeutic alternatives). In some cases, after careful risk evaluation, the MoH may expedite evaluation of drugs that are in shortage to allow the distribution of a drug with relatively minor defects, such as inappropriate labeling or packaging, etc. Cross-contamination of midazolam with the cytotoxic agent amsacrine, described above in this manuscript, is an excellent example of risk management by the Pharmaceutical Division. In another case, similarly to the decision taken by the FDA, a product containing glass particles was allowed to be distributed into the market accompanied by a filter and a DHCP letter instructing the HCP to use the filter to remove potential particles from the product.

According to Procedure 104, it is the responsibility of MoH to notify the public regarding the forthcoming shortage. Furthermore, the MoH actively seeks alternative supply sources of the drug in shortage, to import either a registered or a non-registered drug. Israeli legislation allows for importation of non-registered drugs from authorized countries under article 29 C of the Pharmacist Regulations—Medicinal Products. This allows for a quick and effective way of obtaining drug supply in shortage.

Another step taken by MoH once a shortage is imminent is to instruct the MAH to control more adequately the distribution of the drug in shortage, preventing excess purchase of large quantities by some of the customers and enabling all the stakeholders to receive appropriate restricted drug quantities. An additional strategy may be to instruct physicians, to prescribe the remaining product to defined patient populations and for specific clinical situations, where no appropriate alternatives are available, thus switching or initiating the remaining patients to other therapeutics alternatives.

The presence of a generic drug is not always a guarantee for preventing DSs. The manufacturer may not have the flexibility to increase drug production to meet the national demand in a timely manner, especially at short notice. The MAH of an imported alternative may be unable to increase the supply of the drug into the country due to lack of finished product, previous commitments to other customers and delivery schedules at the manufacturing facility.

In the effort to reduce the number of DSs occurring in Israel each month, the MoH has recently published Procedure 120 which introduces an expedited pathway for authorization of well-established/grandfather drugs. Such drugs were usually registered in Israel in the past or are similar to products registered and marketed for more than 10 years in authorized countries and are imported to Israel under article 29 C. This procedure is currently being evaluated for its impact on reducing the number of non-registered drugs imported to Israel.

One of the causes for cessation of drug distribution in Israel is price reductions. The pricing reference model in Israel is based on overseas reference prices of drugs as well as currency exchange rates. Following the MoH decision on the reference price, HMOs, using an effective tender system may reduce the price even further. This pricing system may be problematic especially with old drugs and small pharma companies. Reference prices for old drugs may be so low that any further decrease makes the production and/or distribution of these important drugs non feasible. Additionally, small local pharma companies may find it difficult to cope with drug suppliers from abroad unwilling to accept the designated price. This challenge has generated important discussions within the Ministry on ways to preserve the availability of such drugs on the market. One of the strategies chosen to overcome this was to suspend any further reductions in drugs prices below 17 new Israeli shekels. The Pharmaceutical Division’s role as an important stakeholder overseeing DSs was an essential factor preventing further reductions below this price.

Finally, as part of Israel preparedness to emergency scenarios (war, earthquake and other natural disasters), it was decided to instruct all MAHs to hold at any time at least 1 month’s supply of all registered drugs in Israel. This strategy was recently published and is monitored and enforced. This requirement is now applicable to both registered drugs and new molecules under evaluation for registration. HMO’s and medical institutions are obliged to ensure a 1 month’s supply of non-registered drugs which are part of the National Health Basket and are imported into Israel under article 29 C of the Pharmacist Regulations—Medicinal Products.

## Conclusions

In this paper, we have described the topic of DSs in Israel, with use of statistics for years 2013–2015. We have analyzed the etiology of DSs and utilized selected examples and the actions taken in their management.

Despite all the steps taken by the Israeli MoH to prevent and manage DSs, their number continues to rise annually. As described in this paper, numerous steps are being taken in the management of DSs, including early notification, working closely with the MAH, preservation of prices for grandfather drugs, procedure 120 and the request for no less than 1 month drug supply in Israel at any time. In order to prevent the selection of one supplier/brand by HMOs due to generic/generic tenders, it was even considered to instruct the HMOs to maintain two suppliers for certain critical drugs, thus limiting the possibility of sole drug supplier on the market.

The MoH periodically publishes a call for manufactures and importers to apply for registration of old well established (grandfather) drugs currently imported under article 29 C. This call was positively taken by potential MAHs.

The MoH preemptively takes measures to overcome the challenge of DSs. A designated unit was established to deal with this ongoing and important task and all DSs are publicly available on the unit’s website. In spite of the time and efforts invested, this problem is yet to be solved. While cooperating with the MAHs is a key factor for this process, currently, there are no deterring measures set in legislation, including financial constraints for any MAH not complying with the requirements set by the MoH. Introducing these deterring measures could serve to minimize the frequency of DS.

DSs pose significant public health hazard worldwide. Early notification and open dialog between the MAH and the regulator is essential for preventing and mitigating the impact of shortages. Further collaboration of all stakeholders, including international stakeholders, is required to overcome these substantial challenges.

## Abbreviations

API: Active Pharmaceutical Ingredient
ATC: Anatomical therapeutic chemical
BIAC: Business and Industry Advisory Committee
CPP: Certificate of pharmaceutical Product
DHCP: Dear Health Care Provider
EMA: European Medicine Agency
EU: European Union
FDA: Food and Drug Administration
FDASIA: Food and Drug Administration Safety and Innovation Act
GMP: Good Manufacture Principles
HMO: Health Management Organization
ICU: Intensive Care Unit
INN: International Nonproprietary Name
MAH: Marketing Authorization Holder
MoH: Ministry of Health
NIS: New Israeli Shekel
OECD: Organization of Economic Cooperation and Development
SOP: Standard Operating Procedure
US: United States
